# In Silico Study of Cell Surface Structures of *Parabacteroides distasonis* Involved in Its Maintenance within the Gut Microbiota

**DOI:** 10.3390/ijms23169411

**Published:** 2022-08-20

**Authors:** Jordan Chamarande, Lisiane Cunat, Corentine Alauzet, Catherine Cailliez-Grimal

**Affiliations:** 1 Stress Imunnity Pathogens (SIMPA), Université de Lorraine, F-54000 Nancy, France; 2 CHRU de Nancy, Service de Microbiologie, F-54000 Nancy, France

**Keywords:** gut microbiota, *Parabacteroides distasonis*, capsular polysaccharide, fimbriae, pilus, O-antigen, pathogenicity, probiotic, comparative genomics

## Abstract

The health-promoting *Parabacteroides distasonis*, which is part of the core microbiome, has recently received a lot of attention, showing beneficial properties for its host and potential as a new biotherapeutic product. However, no study has yet investigated the cell surface molecules and structures of *P. distasonis* that allow its maintenance within the gut microbiota. Moreover, although *P. distasonis* is strongly recognized as an intestinal commensal species with benefits for its host, several works displayed controversial results, showing it as an opportunistic pathogen. In this study, we reported gene clusters potentially involved in the synthesis of capsule, fimbriae-like and pili-like cell surface structures in 26 *P. distasonis* genomes and applied the new RfbA-typing classification in order to better understand and characterize the beneficial/pathogenic behavior related to *P. distasonis* strains. Two different types of fimbriae, three different types of pilus and up to fourteen capsular polysaccharide loci were identified over the 26 genomes studied. Moreover, the addition of data to the *rfbA*-type classification modified the outcome by rearranging *rfbA* genes and adding a fifth group to the classification. In conclusion, the strain variability in terms of external proteinaceous structure could explain the inter-strain differences previously observed of *P. distasonis* adhesion capacities and its potential pathogenicity, but no specific structure related to *P. distasonis* beneficial or detrimental activity was identified.

## 1. Introduction

Gut microbiota (GM) is now considered as a new organ system mainly due to the microorganisms’ specific biochemical interaction with their hosts and their systemic integration into the host biology [1,2]. Bacteria that are predominant in the GM are mainly defined by anaerobic bacteria part of the *Firmicutes* and *Bacteroidetes* phyla [3]. Advances in sequencing methods have facilitated the characterization and understanding of the contribution of the GM to the host well-being, which is now indisputable. In fact, it is now well-defined that the cooperation between the GM and its host is essential to regulate the development and function of the immune, metabolic and nervous system. In turn, one of the major roles of the immune system is to control and maintain its relationships with the GM. The intestinal microbiota, in addition to contributing to the development of the immune system and to intervene into host metabolic and nervous function, also creates a protective barrier against external pathogens and participate in maintaining the structure and integrity of the gastrointestinal tract [4,5,6]. In the long run, the GM can modulate host behavior and nervous system function through dynamic and bidirectional communication along the gut–brain axis [7].

Although mechanisms underlying host–microbiota interactions are not fully described, it is now well-established that cell surface molecules and structures of the GM play a key role in such relationships via conserved microbe-associated molecular patterns (MAMPs) that will be recognized by pattern recognition receptors (PRRs) of immune system cells, including Toll-like receptors (TLRs). An interaction between MAMP and TLR will then initiate the immune response if the MAMP is identified as pathogenic [8,9]. The study of secreted and surface molecules of microbiota members is also fundamental for their involvement in the establishment of species in the versatile and competitive environment of the gut and their key role as a potential virulence factor [10]. Among cell surface markers are capsular polysaccharide (CPS), fimbriae and pili, all well-described for their crucial role in microorganism colonization of the host epithelium.

In Gram-negative anaerobic bacteria, various systems have been described for each of these cell surface markers, including the CPS of *Bacteroides fragilis*, the fimbriae system (Fim) of *Porphyromonas gingivalis*, the type V pilus system (Mfa) of *P. gingivalis* and *Bacteroides thetaiotaomicron* and the immunogenic component of lipopolysaccharide (LPS); O-antigen (Rfb) is well-described in the facultative anaerobic *Escherichia coli* [11,12,13,14,15].

Among the gut microbiota members is *Parabacteroides distasonis*, a Gram-negative bacterium strictly anaerobe belonging to the *Tannerellaceae* family within the *Bacteroidetes* phylum. This bacterial species, part of the *core microbiome*, has recently received a lot of attention, showing beneficial properties for its host. In fact, although strain-dependent, *P. distasonis* display anti-inflammatory/cancer properties and activities on decreasing weight gain, hyperglycemia and hepatic steatosis in *ob*/*ob* and high-fat diet-fed mice [16,17,18]. The importance of *P. distasonis* membrane in these disease treatments has been pointed out in numerous studies. Notably, it has been shown to largely suppress production of pro-inflammatory cytokines in obese animal models [19] and induce apoptosis in colon cancer cell lines, suggesting anti-inflammatory and anti-cancer effects [20]. The membrane components of *P. distasonis* have also been reported to decrease the severity of gut inflammation in the non-immunocompromised mouse models that had induced acute and chronic colitis [21]. Many studies have highlighted these abilities to promote *P. distasonis* as a new potential biotherapeutic product [22,23,24]. In our previous work, we explored *P. distasonis* capacities related to its maintenance within the digestive tract and the electrokinetic properties of its cell peripheral regions to provide a first qualitative picture of its surface structure [25]. This work evidenced a strain-dependent ability to adhere and to form a biofilm related to the putative presence of cell surface structures such as CPS, fimbriae, pili or capsule.

Although numerous studies described the beneficial aspects of *P. distasonis* or its ability to colonize the intestine, few explore mechanisms behind these aptitudes. Moreover, while *P. distasonis* is strongly recognized as intestinal commensal specie with benefits for its host, several studies displayed controversial results, showing *P. distasonis* as an opportunistic pathogen [26,27,28,29]. In this study, we investigated the cell surface structures of *P. distasonis* that may influence host–*P. distasonis* crosstalk and play an essential role in its maintenance and stability within the GM. We reported gene clusters potentially involved in the synthesis of capsule, fimbriae-like and pili-like outer membrane structure and applied the new *rfbA*-typing classification on 26 genomes of *P. distasonis* including 13 new clinical strains (CS) in order to investigate its maintenance within the digestive tract and its potential pathogenicity [30]. In this study, the designation “pilus” is used to describe the external cell surface structure originating from the “minor fimbriae” Mfa system [31,32]. “Fimbriae” refer to structures arising from the Fim system. However, the use of this designation does not mean that Mfa structures are minor and short in comparison with Fim fimbriae [33]; rather, it serves to better clarify the origin of the external appendages described.

## 2. Results

### 2.1. P. distasonis Genomes Characterization

**Figure 1:**
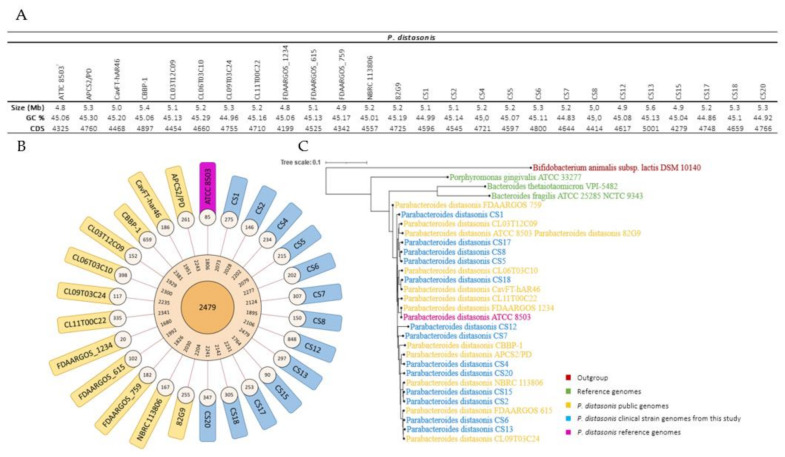
*P. distasonis* genomes characterization. (**A**) General features of *P. distasonis* genomes used in this study. (**B**) Graphical representations of the pangenome characteristics. From the center outward: core, dispensable and specific genome. (**C**) Phylogenetic analysis of 26 strains of *P. distasonis*. *B. thetaiotaomicron* VPI-5482^T^ [39], *P. gingivalis* ATCC 33277^T^ [40] and *B. fragilis* ATCC 25285^T^ [41] were added as reference genomes used in this study. *Bifidobacterium animalis* subsp. *lactis* DSM 10140^T^ was used as outgroup genome.

Thirteen nonredundant *P. distasonis* CS were isolated (Table 1) by the Clinical Microbiology Laboratory of the University Hospital of Nancy, France, and sequenced using Illumina technology. All genomes were then integrated in the Microbial Genome Annotation and Analysis Platform (MaGe) in addition to 13 other public *P. distasonis* genomes (Figure 1).

The length of *P. distasonis* CS genomes range from ~4.8 to 5.6 Mb with an average GC content of 45.00% and a percentage of protein coding density of approximately 91.00%. The pan-genome analysis revealed 2479 functional genes presented in all strains (core-genome), between 1680 and 2479 genes presented in at least two strains function (dispensable genomes) and an average of 253 genes specific to one strain (specific genomes).

The evolutionary relationships among these strains were then investigated by constructing a phylogenetic tree based on the pairwise distances using a neighbor joining algorithm (MaGe).

The tree revealed a partial evolution of *P. distasonis* strains and some similarities notably with FDAARGOS_1234 and ATCC 8503^T^ genomes that appear to be relatively closed. This genome similarity is notably highlighted by the poor specific genomes of both strains.

### 2.2. Identification of P. distasonis Genes Potentially Involved in Capsule, Fimbriae-like and Pilus-like Synthesis

In order to determine the potential presence of capsule, fimbriae or pili at the surface of *P. distasonis*, reference genes involved in their synthesis were selected from *B. fragilis* (gut), *B. thetaiotaomicron* (gut) and *P. gingivalis* (oral cavity), as three strictly anaerobe Gram-negative bacteria part of the Bacteroidetes phyla, and referenced as opportunistic pathogens [39,40,41]. Indeed, *B. fragilis* is well-known for its numerous divergent polysaccharides loci all starting by genes designated as UpxY and UpxZ families, where x goes from a to h depending on the locus. *upxY* genes are transcriptional antitermination factors essential to the CS synthesis, while *upxZ* genes inhibit their secretions [11]. *P*. *gingivalis*, for its part, is well described for its proteinaceous, filamentous appendages at its surface including fimbriae and pili, synthesized through the Fim (fimA-E) and Mfa (mfa1-5) systems, respectively [15]. A similar Mfa system including only *mfa1* and *mfa2* has also been described in *B. thetaiotaomicron* [13].

Synteny analysis of reference genes on *P. distasonis* genomes revealed a set of genes whose function possibly approaches that of the reference genes. To refine the search, only genes with an automatic functional assignation linked to the synthesis of the sought structures were listed (Table 2).

No result was found for *up(a-g)Y* and *up(a-h)Z*, while 15 genes from 15 distinct strains were referred to as potentially *uphY*-like with homologies ranging from 33.10% to 36.90%. Concerning the fim gene cluster, although homologies are relatively low (from 22.50% to 27.10%), all reference genes possess a synteny in at least one genome of P. distasonis with an auto-assigned function related to the fimbriae synthesis. The synteny analysis between the *mfa* cluster of *B. thetaiotaomicron* VPI-5482^T^ and *P. distasonis* genomes revealed only one positive result for *B. thetaiotaomicron mfa2*, while the *mfa* gene cluster of *P. gingivalis* ATCC 33277^T^ permitted the listing of multiple genes for *P. gingivalis mfa1, mfa2* and *mfa4*. No result was found for *P. gingivalis mfa3* and *mfa5* genes.

Multiple sequence alignments of the listed genes (Figure S1) revealed either the conservation of one sequence (*fimB*-like, *fimC*-like, *fimD*-like, Bt *mfa2*-like, Pg *mfa1*-like and Pg *mfa4*-like) or the presence of two distinct sequences (*uphY*-like 1/2, *fimA*-like 1/2, *fimE*-like 1/2 and Pg *mfa2*-like 1/2).

#### 2.2.1.* P. distasonis* Gene Cluster Potentially Involved in Capsule Synthesis

**Figure 2:**
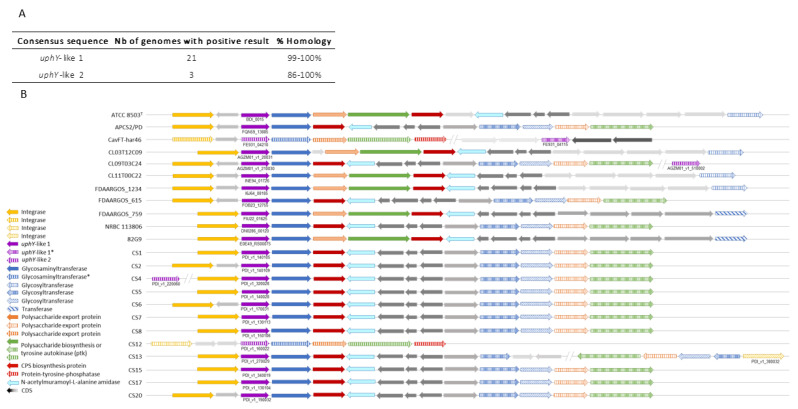
Identification of *upxY*-like gene clusters on *P. distasonis* genomes. (**A**) BLAST of *upxY*-like consensus sequences against *P. distasonis* genomes. (**B**) Syntenic analysis of *P. distasonis* capsular polysaccharide gene clusters centered on *uphY*-like 1 gene. Vertical and diagonal striped arrows refer to genes having a high homology and sharing synteny with reference gene. (*) indicate genes with low homology but sharing synteny over *P. distasonis* genomes. CDS: coding sequences are represented by gray arrows.

BLAST of the consensus sequences *uphY*-like 1 against *P. distasonis* genomes revealed genes with high similarity (from 99% to 100%) in 21 of the 26 studied genomes (Figure 2A). Among these genes, 15 are from the syntenic analysis while 6 are from BLAST. These last six sequences were not found during the syntenic analysis probably due to variations in their genomic organizations. On the contrary, the *uphY*-like 2 was identified in only three genomes with still an important sequence conservation (from 86% to 100%). Each *uphY*-like genomic region was then analyzed to allow the discovery of very conserved regions with a high gene homology (Figure 2B). Among them are genes linked to the CPS synthesis including glycosyltransferase, polysaccharide export, polysaccharide biosynthesis and CPS biosynthesis genes. Each CPS cluster is also composed of downstream gene encoding an integrase. The three genes similar to *uphY*-like 2 were analyzed and integrated at the syntenic analysis. The genomic environment of *uphY*-like 2 appear to be relatively close to the first loci identified with *uphY*-like 1, including an integrase, a glycosyltransferase, a polysaccharide export, a polysaccharide biosynthesis and a CPS biosynthesis gene, too.

Specific research on *P. distasonis* ATCC 8503^T^ CPS loci genomes allowed us to find a 14th CPS loci, in addition to the 13 already identified [42]. All CPS loci were then explored on other *P. distasonis* genomes (Table 3, gene details in Table S1). Among the 26 *P. distasonis* genomes, only ATCC 8503^T^ and FDAARGOS_1234 possess the 14 CPS loci identified. Loci 3, 6, 9, 13 and 14 are shared between all *P. distasonis*, while only few strains possess loci 10, 11 and 12. Loci 7 and 8 are also conserved over genomes, but important intra-variations have been identified within these loci. Moreover, not all gene loci are different: 2 and 8 show high gene sequence conservation with a similar *upxY*-like gene. Locus 5 appear to be relatively close to 2 and 8 too, but with more variations. In the same way, the locus 4 shows some similarities with 2, 5 and 8 but has a different *upxY*. On the contrary, locus 13 possesses a similar *upxY* to 2, 5 and 8 but a different locus. Loci 3, 6, 7 and 1, 11, 12 also display similarities, especially between 6, 7 and 11, 12. Locus 1, although close to 11 and 12, presents a distinct *upxY*. Moreover, the conserved part of locus 9 does not always seem to be the one involved in the CPS synthesis.

In addition to *upxY* genes, several of these CPS loci contain a phage insertion (N-acetylmuramoyl-L-alanine amidase, homolog of phage T7 lysozyme) that may modulate its expression (Table 3). Among them, CPS loci 1 and 13 of the 26 *P. distasonis* genomes all harbor these insertions. For CPS loci 1, this inserted segment (light blue arrows in Figure 2) is oriented in the opposite direction to *upxY*-like gene downstream of the CPS biosynthesis genes (red arrows in Figure 2).

#### 2.2.2. *P. distasonis* Gene Cluster Potentially Involved in Fimbriae-like Synthesis

**Figure 3:**
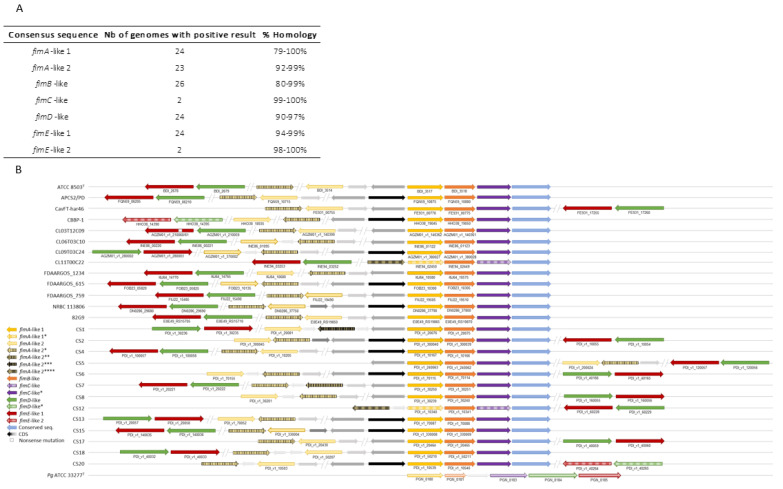
Identification of *fim*-like gene clusters on *P. distasonis* genomes compared to *P. gingivalis* cluster. (**A**) BLAST of *fim*-like consensus sequences against *P. distasonis* genomes. (**B**) Syntenic analysis of *P. distasonis* fimbriae gene clusters centered on *fimA*-like 1 gene. Vertical striped arrows refer to genes having a high homology and sharing synteny with reference gene. (*) indicate genes with low homology but sharing synteny over *P*. *distasonis* genomes. CDS: coding sequences are represented by gray and black arrows. Horizontal striped arrows are used for the Fim cluster of the reference genome: *P. gingivalis* ATCC 33277^T^.

Almost all the *fim*-like genes investigated have been identified in the 26 *P. distasonis* genomes (Figure 3). The few genes not found by BLAST have been highlighted in the syntenic analysis showing *fimA-E* on every *P. distasonis* genome. Notably, BLAST of *fimC*-like allowed the identification of another *fimC*-like gene possessed by 24 of the 26 studied genomes. The identified *fim*-like gene cluster is composed of a various gene blocks, including one main block of four genes (*fimA*-like, *fimB*-like and *fimC*-like); a second block of two genes (*fimD*-like and *fimE*-like) that are always together but not located in the same region as *fimA-C*; and several genes showing a slight homology but a synteny with *fimA*-like 2, which are located sometimes in and sometimes out of the main block of genes. One nonsense mutation was found on the *fimE*-like gene of the CL03T12C09 that probably avoid its synthesis. Compared to the *P. gingivalis fim* cluster, whose genes all follow each other, *P. distasonis fim*-like cluster appeared to be relatively close in terms of organization, with only *fimD*-like and *fimE*-like displaying a different location.

#### 2.2.3. *P. distasonis* Gene Cluster Potentially Involved in Pili-like Synthesis

**Figure 4:**
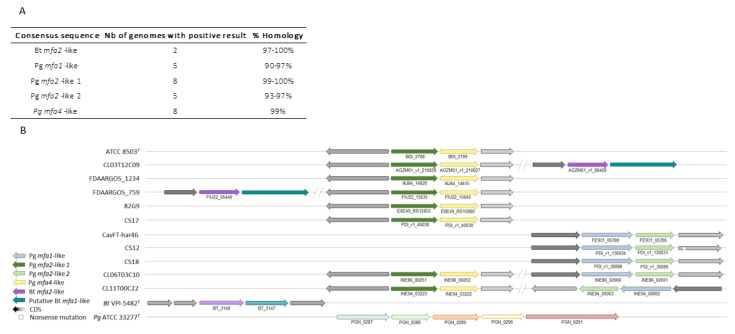
Identification of pili-like gene clusters on *P. distasonis* genomes compared to *P. gingivalis* and *B. thetaiotaomicron* cluster. (**A**) BLAST of *mfa*-like consensus sequences against *P. distasonis* genomes. (**B**) Syntenic analysis of *P. distasonis* pilus gene clusters centered on *fimA*-like 1 gene. CDS: coding sequences are represented by gray arrows. Horizontal striped arrows are used for the Mfa cluster of the reference genomes: *B. thetaiotaomicron* VPI-5482^T^ and *P. gingivalis* ATCC 33277^T^.

Homologue sequences of Bt *mfa2*-like genes were found in only two *P. distasonis* genomes, while Pg *mfa1*-like/Pg *mfa2*-like 2 and Pg *mfa2*-like 1/Pg *mfa4*-like genes were found on five and eight genomes, respectively (Figure 4A). Interestingly, the five genomes containing Pg *mfa1*-like gene correspond to the five genomes holding Pg *mfa2*-like 2. In the same way, the eight genomes positive to the BLAST are the same for Pg *mfa2*-like 1 and Pg *mfa4*-like genes. The syntenic analysis of Bt *mfa2*-like gene (Figure 4B) revealed a conserved gene downstream of Bt *mfa2*-like gene showing similarities with Bt *mfa1*, identified as putative Bt *mfa1*-like gene. Some strains harbor several *mfa*-like clusters, such as putative Bt *mfa1*-like/Bt *mfa2*-like + Pg *mfa2*-like 1/Pg *mfa4*-like genes or Pg *mfa1*-like/Pg *mfa2*-like 2 + Pg *mfa2*-like 1/Pg *mfa4*-like genes.

### 2.3. rfbA Classification and Investigation

**Figure 5:**
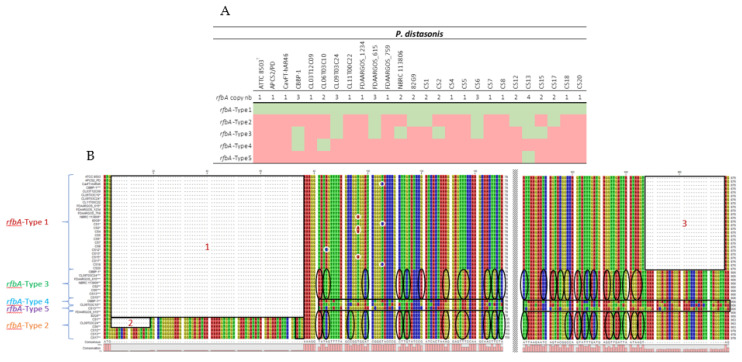
Classification and characterization of *rfbA* genes of *P. distasonis*. (**A**) *rfbA* copy number and classification of each *P. distasonis* strain. Color code: presence (green) or absence (red) of the *rfbA* gene of the indicated type. (**B**) *rfbA*-type nucleotide sequences and gaps analysis. Gaps are framed and numerated from 1 to 3. *rfbA*-type 1 single-nucleotide polymorphism are surrounded in white. Variation of *rfbA*-type 2 and 3 from *rfbA*-type 1 are highlighted by black circles. *rfbA*-type 4 and 5 are framed in black due to the important number of variations compared to the *rfbA*-type 1.

In order to determine the potential pathogenicity of *P. distasonis*, the new *rfbA*-type classification was applied to the 26 studied genomes (Figure 5A, gene details in Table S2). The addition of new data modified the classification. A fifth group was identified and the previous gene repartition changed, notably with the presence of a *rfbA*-type 1 gene in all the 26 *P*. *distasonis* strains. In order to better understand the variation between each *rfbA*-type gene, the multiple sequence alignment of all the *rfbA* genes was explored (Figure 5B). The analysis revealed the presence of three gaps, two in 5*′* and one in 3*′*. The *rfbA*-type 1 seems to be characterized by the presence of gaps 1 and 2, leading to a shorter *rfbA* sequence (876 nucleotides) with some point mutation observable. The *rfbA*-type 2, in addition to being characterized by the gaps 1 and 2, shows specific variations compared to the *rfbA*-type 1. Interestingly, a start codon ATG is observable in position 73 of every *rfbA*-type 2 gene that could lead to the suppression of the gap 1. The *rfbA*-type 3 is also identified by the gap 1 and variations from *rfbA*-type 1 that are relatively closed to *rfbA*-type 2. The gap 1 is also present for the types 4 and 5 which, however, display very unique sequences.

### 2.4. Implication of P. distasonis Cell Surface Structures in Its Potential Pathogenicity

All the data generated in this study were compiled in order to determine the implication of *P. distasonis* cell surface markers in its potential pathogenicity (Table 4). Strains were classified as commensal (ATCC 8503^T^ and NBRC 113806) or potential pathogens (CavFT-hAR46 and CS1-20 except CS6) on the basis of the health status of their original host (based on the isolation source of each strain, Table 1), and as beneficial or detrimental based on the literature [16,20,26,27,36]. The comparison of outer membrane structure from both categories does not bring to the fore any specific structure. Indeed, all the external structures harbored by the potential pathogen strains are identified in at least one of the commensal strains. In the same way, all structures absent from the surface of commensal strains are not systematically carried by the potential pathogens.

## 3. Discussion

The human GM and its trillion of bacteria are now well-known for their commensal and symbiotic relationships with the host. One of the GM members is *P. distasonis*, a Gram-negative anaerobe part of the *core microbiome*. While a large number of studies promotes this species as a new potential biotherapeutic product due to its multiple benefits provided to its host, controversial results have identified it as an opportunistic pathogen [22,23,24,26,27,28,29]. Although there is still a lot to understand about the mechanisms involved in the GM–host interaction, the implication of cell surface structures of GM members is now well-defined [9,43,44]. In the present study, we investigated cell surface structures of 26 *P. distasonis* genomes in order to better understand its maintenance within the digestive tract and its potential virulence. Among the 26 genomes, 13 new clinical strain genomes of the member of the distal gut microbiome *P. distasonis* were sequenced and computed on the MaGe platform. The general features of new genomes were very similar to other *P. distasonis* genomes already available, with an average size of 5.2 Mb and a core-genome of 2479 CDS. The phylogenetic analysis did not highlight any special difference between CS genome from this study and other *P. distasonis* genome with a homogenous distribution of CS genomes over the tree.

A previous investigation of CPS loci revealed the presence of the UpxY regulator on *P. distasonis* ATCC 8503^T^ genome, leading to the identification of 13 putative CPS loci over its genome [42]. In our study, a 14th putative locus was identified on the ATCC 8503^T^ genome. Although well conserved, not all 14 CPS loci are conserved over the 26 *P. distasonis* strains investigated in this study. Surprisingly, any of the *upxY* genes identified seem to be coupled with a *upxZ* regulator genes. However, if UpxY positively regulates *B. fragilis* CPS synthesis by preventing premature transcription termination in the untranslated region, UpxZ is indispensable to limit production of multiple CPSs, as described in *B. fragilis* and *B. thetaiotaomicron* [45,46]. Consequently, *P. distasonis* surface polysaccharide seems to result in the combination of multiple CPS loci whose expression is potentially controlled by inversions of the promoter region, leading to phase variable synthesis [47]. Th presence of phage insertions within several of these CPS loci may also modulate its expression. In addition, *P. distasonis* strains do not all display the same number of CPS loci and can also have sequence variations over the loci, emphasizing the strain-dependent nature of *P. distasonis* CPS.

In addition to external polysaccharides, another proteinaceous surface structure involved in host–microbiota interaction is the fimbriae. One of the most described fimbriae organization is the *P. gingivalis* Fim system strongly identified as a virulence factor [15]. A previous study identified an analogous typical pilin encoding operon on the *P. distasonis* ATCC 8503^T^ genome [48]. In our work, almost all the *fim*-like genes investigated were identified in the 26 *P. distasonis* genomes, revealing the important conservation of a gene cluster involved in the fimbriae-like synthesis. Among these clusters, two distinct type of fimbriae were identified. The first one is present on 24 of the 26 studied genomes and seems to be conserved, while the second one is only harbored by two genomes. Both clusters are composed of *fimA-B-C-D-E*-like genes with some variations, including the putative presence of other *fimA*-like genes through the Fim clusters or different gene sequences such as the *fimD-E* of CBBP-1 and CS20 strains that display low similarity with others *fimD-E*. Fimbriae do not necessarily mean pathogenicity by contributing to host epithelium colonization, thus forming a protective barrier against external pathogens and stimulating the host immune system, as recently demonstrated by the recombinant pLA-K88/*Lactobacillus casei* strain [49].

Pili, as well as capsular polysaccharides and fimbriae, are external proteinaceous structures involved in host–GM interaction. *P. gingivalis* that display fimbriae also harbor pili, also called “minor fimbriae” [33]. The Mfa system of *P. gingivalis* involved in the pilus synthesis has also been identified in the gut commensal *B. thetaiotaomicron* [13]. Although partially found, no complete *mfa*-like gene cluster has been identified in the studied *P. distasonis* strains. However, 11 of the 26 genomes possess a pair of genes composed of either Bt *mfa1/mfa2*, Pg *mfa1/mfa2* or Pg *mfa4/mfa2* with *mfa1/mfa4* encoding for an external polymer and *mfa2* involved in the anchoring of the pilus and length regulation of Mfa1. The absence of pilus gene cluster on other genomes could be explained either by a greater diversity of pilus with pili showing important differences from the investigated ones or by the absence of pili on more than half of the studied strains. As for fimbriae, the presence of pili does not necessarily imply pathogenicity. The well-studied probiotic *Lactobacillus rhamnosus* GG and its spaCBA-encoded pili confirmed this by showing multiple benefits for its host despite its proteinaceous heteropolymeric extracellular appendages [50].

**Figure 6:**
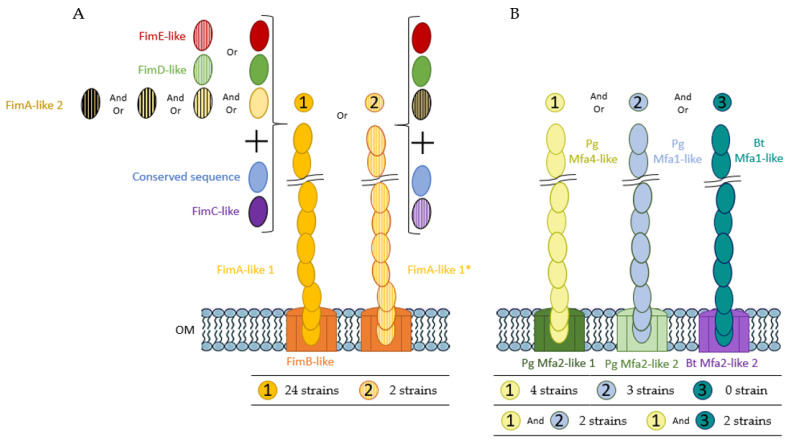
Hypothetical schematic representation of *P. distasonis* (**A**) fimbriae and (**B**) pili from the Fim and Mfa system, respectively. The different type of fimbriae and pili have been identified from 1 to 2 and from 1 to 3, respectively. The table below each structure represents the number of strains harboring the gene cluster encoding the hypothetical structure. The color code corresponds to the syntenic analysis. “Or” indicates that one *P. distasonis* strain can harbor only one of the proteins encoding genes concerned. For example, type 1 Fim cluster of *P. distasonis* contains either *fimE*-like or striped *fimE*-like genes but never both in the 24 identified clusters. “And Or” indicates that one *P. distasonis* strain can harbor one or several of the protein encoding genes. For example, various *fimA*-like 2 genes combinations can be found within type 1 Fim cluster of *P. distasonis*.

The identification of fimbriae-like and pili-like gene clusters allowed the representation of cell surface markers potentially present at the surface of *P. distasonis* (Figure 6). Two distinct fimbriae-like and three pili-like gene clusters have been represented depending on the gene clusters found. The first type of fimbriae (left) is harbored by 24 *P. distasonis* while the second one (right) is harbored by the last two studied strains (CL11T00C22 and CS12). Concerning the pili, four strains (ATCC 8503^T^, FDAARGOS_1234, 82G9 and CS17) harbor only the first type (left), three (CavFT-har46, CS12 and CS18) harbor only the second type (middle) and none harbor only the third type (right). Some strains also presented combination of several pilus: two (CL06T03C10 and CL11T00C22) harbor the first and second pili type and two (CL03T12C09 and FDAARGOS_759) harbor the first and third type.

In order to discriminate strains regarding their LPS, all the *rfbA* genes of the 26 *P. distasonis* genomes have here been referenced, and RfbA-type classification has been applied. As described in Bank et al., 2022 [30], most of the listed *rfbA* genes belong to type I, highlighting the conservation of this LPS type with the ATCC 8503^T^ *rfbA* belonging to the type I, and that was isolated more than 80 years ago. However, the addition of new data modified the classification with the identification of a fifth type and a new repartition of the *rfbA* genes within the five types. The analysis of *rfbA*-type variation revealed the presence of three main gaps and multiple sequence variations that shape the *rfbA*-type organization, including one major gap potentially non-existent due to the presence of a start codon within some *rfbA* gene sequences. Unlike the previous classification, the new *rbfA* gene repartition shows CS possessing type 1 *rfbA* and no specific *rfbA*-type allowing the distinction of CS from other *P. distasonis* strains. Thus, this typing does not seem to be adequate to differentiate pathogenic from non-pathogenic strains.

The comparison of outer membrane structures from commensal to potential pathogenic strains does not allow the identification of specific surface markers responsible for the putative pathogenicity of *P. distasonis*. The inter-strain variability observed for *P. distasonis* properties and potential pathogenicity could be explained by the association of all differences observed in this study, including the presence/absence of cell surface markers, loci/clusters organization and gene sequences. These variations are correlated with the phylogenetical analysis where, for example, *P. distasonis* ATCC 8503^T^ and FDAARGOS_1234 strains, which are genetically close, display the exact same external structures. In the same way, FDAARGOS_759 and CL09T03C24 that are the most genetically different strains appear to differ from each other in the presence/absence of seven outer structures. Moreover, the synthesis of these external structures seems to depend on numerous factors, including genetic regulators themselves potentially contingent on environment conditions in which bacteria are evolving [47,51,52]. Thus, one plausible response to the pathogenic effects of *P. distasonis* is the involvement of other mechanisms than its CPS, pili, fimbriae or LPS/O-antigen membrane fractions and to the dissemination of this species from the GM to sterile sites, as an opportunistic pathogen.

Concerning *P. distasonis* maintenance within the GM, the presence of such external proteinaceous structures could explain its ability to adhere and persist in this complex and competitive environment. These results are consistent with our previous study that illustrates the adhesion and biofilm formation capacity of the 13 *P. distasonis* [25]. Although all the strains were able to adhere, an inter-strain variation was observable. These differences could be explained by a different shape of the external surface of the strains. Interestingly, the CS12 that displays the lowest adhesion capacity is also the only CS strain that does not harbor the type 1 fimbriae gene cluster identified in this study. This result could highlight the potential involvement of the type 1 fimbriae in the maintenance of *P. distasonis* within the GM. However, it does not seem that there is a link between the presence of a special cell surface marker for the higher adhesion or biofilm abilities. In fact, the CS1 that displays the most important adhesion capacity does not show a specific cell surface appendage that could explain this adhesion capacity. In the same way, the CS8 that has the strongest biofilm formation capacity does not show a particular cell surface structure explaining its greater capacity.

In conclusion, this work permitted the identification of several gene clusters involved in the capsule, fimbriae and pili synthesis. The presence or absence of these cell surface structures coupled with variations in gene sequences could explain *P. distasonis* maintenance within the GM and the inter-strain variability observed for its beneficial capacities and potential pathogenicity. However, no specific external cell surface structure that could explain *P. distasonis* behavior was identified. This study provides a better comprehension of the preservation of *P. distasonis* through the human gut and tools to better understand and characterize the beneficial/pathogenic behavior related to *P. distasonis* strains.

## 4. Materials and Methods

### 4.1. Whole-Genome Sequencing

The genomic DNA of the 13 CS of *P. distasonis* was extracted by the QiaAmpDNA MiniKit (Qiagen, Courtaboeuf, France). De Novo Microbial Genome Sequencing using Illumina technology was used to sequence the 13 CS of *P. distasonis* (Eurofins Genomics, Ebersberg, Germany). All genomes were integrated in the MaGe platform [53] (v3.15.3; The LABGeM, CEA/Genoscope and CNRS UMR8030).

### 4.2. Genome Data Used

In silico analyses of cell surface structures were performed on the 13 CS of *P. distasonis* and 13 public genomes available on the MaGe platform.

### 4.3. Pan and Core-Genome Analysis

The pan and core-genome of *P. distasonis* were calculated with the Pan/Core-Genome tool of MaGe, based on MicroScope gene families (MICFAM) which are computed using an algorithm implemented in the SiLiX software. The following were used as stringent parameters: 80% amino acid identity and 80% alignment coverage.

### 4.4. Phylogenetic Analysis

*P. distasonis* whole-genome sequences were used to determine the phylogenetic relationship among the isolates and public databases. Reference genomes (*P. gingivalis* ATCC 33277^T^, *B. thetaiotaomicron* VPI-5482^T^ and *B. fragilis* ATCC 25285^T^) used in this study were added to the tree to demonstrate their closeness with *P. distasonis*. The phylogenetic tree was computed on MaGe using the Genome Clustering tool and reworked on the Interactive Tree Of Life online tool [54] (iTOL).

### 4.5. Comparative Genome Analysis

In order to determine the potential presence of fimbriae, pili and/or capsular polysaccharides at the surface of *P. distasonis*, reference genes involved in their synthesis were selected from species related to *P. distasonis* (Table 5).

Synteny enabled us to identify the conservation of homologous genes and gene order between genomes of different strains or species. Synteny blocks between references and *P. distasonis* genomes were investigated using the Genome Browser/Syntonome tools of MaGe and allowed the selection of a pool of genes potentially involved in the synthesis of the targeted structures. To reduce the number of *P. distasonis* genes and refine the search, only genes with an auto-assignation function related to the synthesis of the sought element were preserved and listed. The automatic functional assignation of MaGe follows an algorithm based on homologous relations with model organisms and completion of gene editor (gene name, product, EC numbers, roles…) using various programs or databases (RefGen, SwissProt, UniFIRE, TrEMBL…).

Multiple sequence alignments of each pool of genes related to each reference gene were then performed using CLC Viewer 8.0 to obtain one or several consensus sequences related to each reference gene.

Consensus sequences were then used for BLAST investigation against the 26 *P. distasonis* genomes using the Blast and Pattern Searches tool of MaGe.

Matching genes were then used to generate a syntenic block analysis between *P. distasonis* genome for each cell surface structure studied.

### 4.6. rfbA-Type Determination and Analysis

In order to determine the *rfbA*-type genes of the latest sequenced *P. distasonis* genomes, the classification method recently described by Bank et al. was used [30].

*rfbA* genes of *P. distasonis* were first referenced and aligned using CLC Viewer 8.0. The multiple sequence alignment was then used to generate a phylogenetic tree, allowing the classification of new *rfbA* genes.

As the *rfbA*-type genes obtained in this study were different from the previous classification, analyses of nucleotide sequences and gaps of distinct *rfbA*-type genes were performed in order to determine and better understand the variation between each type.

## Figures and Tables

**Table 1:** *P. distasonis* strain isolation.

	Strain	Type of Sample	Host Status	Isolation Date	Isolation Country	References
***Parabacteroides distasonis***	ATCC 8503^T^	Human feces	Apparently normal	1933	USA	[34]
	APCS2/PD	Human feces	Unknown	2017	Ireland	NCBI
	CavFT-hAR46	Human intramural gut wall	Severe Crohn’s disease	2019	USA	[35]
	CBBP-1	Feces	Unknown	Unknown	Unknown	[36]
	CL03T12C09	Unknown	Unknown	Unknown	Unknown	NCBI
	CL06T03C10	Human feces	Unknown	2009	USA	[37]
	CL09T03C24	Unknown	Unknown	Unknown	Unknown	NCBI
	CL11T00C22	Human feces	Unknown	2009	USA	[37]
	FDAAROS_1234	Unknown	Unknown	Unknown	Unknown	NCBI
	FDAARGOS_615	Human feces	Unknown	Unknown	Unknown	Not Published
	FDAARGOS_759	Human feces	Unknown	Unknown	USA	[38]
	NRBC 113806	Human feces	Normal	Unknown	Unknown	NCBI
	82G9	Human feces	Unknown	Unknown	Japan	NCBI
	CS1	Peritoneal fluid	Peritonitis	2016	France	[25]
	CS2	Peritoneal fluid	Peritonitis	2016	France	[25]
	CS4	Vulvectomy	Vulvar infection	2016	France	[25]
	CS5	Peritoneal fluid	Peritonitis	2016	France	[25]
	CS6	Sterility control of mesenchymal stem cells	Unknown	2016	France	[25]
	CS7	Peritoneal fluid	Peritonitis	2016	France	[25]
	CS8	Blood culture	Bacteremia	2016	France	[25]
	CS12	Bone, sacrum	Osteo-articular infection	2016	France	[25]
	CS13	Peritoneal fluid	Peritonitis	2016	France	[25]
	CS15	Peritoneal fluid	Peritonitis	2016	France	[25]
	CS17	Small intestine collection	Abdominal abscess	2017	France	[25]
	CS18	Abdominal collection	Abdominal abscess	2017	France	[25]
	CS20	Peritoneal fluid	Peritonitis	2017	France	[25]

^T^—type strain in microbiology.

**Table 2:** *P. distasonis* genes sharing synteny with reference genes and auto-assigned as part of CPS, fimbriae or pili synthesis.

Structure	Reference Gene	*Pdist* Strain	Label	Length (aa)	Automatic Assignation of Biological Function	% Homology
**Capsule**	*up(a-g)Y*	No match				
	*up(a-g)Z*	No match				
	*uphY*	CS12	PDI_v1_160022	185	Transcription antitermination protein UpdY	36.90
		CL09T03C24	AGZN01_v1_510002	192	Transcription antitermination protein UpdY	36.00
		CS4	PDI_v1_220060	192	Transcription antitermination protein UpdY	36.00
		FDAARGOS_615	FOB23_12755	179	UpxY family transcription antiterminator	33.72
		APCS2/PD	FQN59_13885	179	UpxY family transcription antiterminator	33.70
		CS2	PDI_v1_140109	179	Transcription antitermination protein UpdY	33.70
		CS5	PDI_v1_140028	179	Transcription antitermination protein UpdY	33.70
		CS6	PDI_v1_170031	179	Transcription antitermination protein UpdY	33.70
		CS8	PDI_v1_150106	179	Transcription antitermination protein UpdY	33.70
		CS15	PDI_v1_340019	179	Transcription antitermination protein UpdY	33.70
		CL03T12C09	AGZM01_v1_20031	179	Transcription antitermination protein UpdY	33.14
		FDAARGOS_759	FIU22_01625	179	UpxY family transcription antiterminator	33.14
		82G9	E0E49_RS00075	179	UpxY family transcription antiterminator	33.14
		CS1	PDI_v1_140105	179	Transcription antitermination protein UpdY	33.10
		CS7	PDI_v1_130113	179	Transcription antitermination protein UpdY	33.10
	*upgZ*	No match				
**Fimbriae**	*fimA*	82G9	E0E49_RS19850	444	fimbrial protein	26.21
		ATCC 8503^T^	BDI_3514	444	putative fimbrial protein precursor	25.99
		CavFT-hAR46	FE931_00755	444	fimbrial protein	25.99
		FDAARGOS_759	FIU22_19490	444	fimbrial protein	25.99
		CS6	PDI_v1_70115	432	Fimbrial protein	25.60
		CS13	PDI_v1_70087	432	Fimbrial protein	25.60
		CL11T00C22	INE94_02450	431	Major fimbrium subunit FimA type-2	25.30
		CS12	PDI_v1_10340	431	Major fimbrial subunit protein (FimA)	25.10
		CS1	PDI_v1_20076	434	Major fimbrial subunit protein type II	24.90
		CS2	PDI_v1_300040	419	Fimbrial protein	24.20
		CS15	PDI_v1_330008	419	Fimbrial protein	24.20
		CS20	PDI_v1_10539	419	Fimbrial protein	24.20
		APCS2/PD	FQN59_10875	419	fimbrial protein	24.10
		CS4	PDI_v1_10167	419	Fimbrial protein	24.10
		CL06T03C10	INE86_01122	420	Major fimbrium subunit FimA type-2	24.00
		CS18	PDI_v1_50210	420	Fimbrial protein	24.00
		CS8	PDI_v1_30239	421	P_gingi_FimA domain-containing protein	23.70
		CS5	PDI_v1_240063	421	P_gingi_FimA domain-containing protein	23.70
		CS17	PDI_v1_20464	421	P_gingi_FimA domain-containing protein	23.70
		FDAARGOS_1234	I6J64_10580	421	fimbrial protein	23.50
		CS7	PDI_v1_30250	437	Fimbrial protein	23.20
	*fimB*	82G9	E0E49_RS19870	303	FimB/Mfa2 family fimbrial subunit	29.90
		CBBP-1	HHO38_19050	303	FimB/Mfa2 family fimbrial subunit	29.90
		CL06T03C10	INE86_01123	303	Fimbrillin-A associated anchor proteins Mfa1 and Mfa2	29.90
		FDAARGOS_1234	I6J64_10575	303	FimB/Mfa2 family fimbrial subunit	29.90
		FDAARGOS_759	FIU22_19510	303	FimB/Mfa2 family fimbrial subunit	29.90
		CS1	PDI_v1_20075	303	Fimbrillin-A associated anchor proteins Mfa1 and Mfa2	29.90
		CS6	PDI_v1_70114	303	FimB/Mfa2 family fimbrial subunit	29.90
		CS12	PDI_v1_10341	305	Fimbrillin-A associated anchor proteins Mfa1 and Mfa2	29.90
		CS13	PDI_v1_70088	303	FimB/Mfa2 family fimbrial subunit	29.90
		CL11T00C22	INE94_02449	305	Fimbrillin-A associated anchor proteins Mfa1 and Mfa2	29.00
	*fimC*	CL11T00C22	INE94_02448	375	Putative fimbrium tip subunit Fim1C	22.50
	*fimD*	CS2	PDI_v1_10054	684	P_gingi_FimA domain-containing protein	26.40
		CS15	PDI_v1_140036	684	P_gingi_FimA domain-containing protein	26.40
		CS12	PDI_v1_60229	685	P_gingi_FimA domain-containing protein	26.10
		CS17	PDI_v1_40059	685	P_gingi_FimA domain-containing protein	26.10
		CS18	PDI_v1_40032	685	P_gingi_FimA domain-containing protein	26.10
		CL03T12C09	AGZM01_v1_210059	684	P_gingi_FimA domain-containing protein	26.02
		CS5	PDI_v1_120056	675	P_gingi_FimA domain-containing protein	25.70
		CS8	PDI_v1_160055	675	P_gingi_FimA domain-containing protein	25.70
		CS4	PDI_v1_100056	677	P_gingi_FimA domain-containing protein	25.10
		CL09T03C24	AGZN01_v1_280002	678	P_gingi_FimA domain-containing protein	24.76
	*fimE*	CL11T00C22	INE94_03253	632	Major fimbrium tip subunit FimE	27.10
		CL06T03C10	INE86_00220	632	Major fimbrium tip subunit FimE	25.30
		CBBP-1	HHO38_14390	688	FimB/Mfa2 family fimbrial subunit	23.01
**Pilus**	Bt *mfa1*	No match				
	Bt *mfa2*	FDAARGOS_759	FIU22_05440	350	FimB/Mfa2 family fimbrial subunit	28.98
	Pg *mfa1*	CS12	PDI_v1_130034	509	Fimbrillin_C domain-containing protein	26.50
		CS18	PDI_v1_30088	509	Fimbrillin_C domain-containing protein	26.50
		CL06T03C10	INE86_02000	392	Minor fimbrium subunit Mfa1	25.40
		CL11T00C22	INE94_00002	509	Major fimbrial subunit protein type IV	25.40
	Pg *mfa2*	CL06T03C10	INE86_02001	329	Minor fimbrium anchoring subunit Mfa2	31.20
		CL11T00C22	INE94_00003	329	Minor fimbrium anchoring subunit Mfa2	31.20
		CS12	PDI_v1_130033	329	FimB/Mfa2 family fimbrial subunit	30.90
		CS18	PDI_v1_30089	329	putative Minor fimbrium anchoring subunit Mfa2	30.40
		FDAARGOS_759	FIU22_15640	300	FimB/Mfa2 family fimbrial subunit	24.32
		82G9	E0E49_RS15860	300	FimB/Mfa2 family fimbrial subunit	24.32
	Pg *mfa3*	No match				
	Pg *mfa4*	FDAARGOS_759	FIU22_15635	463	Mfa1 fimbrilin C-terminal domain-containing protein	20.83
		ATCC 8503^T^	BDI_2708	463	putative outer membrane protein	20.51
		CL03T12C09	AGZM01_v1_210028	463	Fimbrillin_C domain-containing protein	20.51
		82G9	E0E49_RS15855	463	Mfa1 fimbrilin C-terminal domain-containing protein	20.20
	Pg *mfa5*	No match				

aa: amino acid; Bt: *B. thetaiotaomicron*; Pg: *P. gingivalis.*.

**Table 3:** Identification of CPS loci in 26 *P. distasonis* genomes and phage insertion within clusters. Color code: presence (green), partial presence (orange) or absence (red) of the CPS locus by comparison with ATCC 8503^T^ CPS loci. Partial clusters include loci either possessing similar genes compared to ATCC 8503^T^ loci but no *upxY*-like gene or an identical *upxY*-like gene to ATCC 8503^T^ but a different gene locus. ● indicate loci containing phage gene insertions.

		*P. distasonis*																									
		ATTC 8503^T^	APCS2/PD	CavFT-hAR46	CBBP-1	CL03T12C09	CL06T03C10	CL09T03C24	CL11T00C22	FDAARGOS_1234	FDAARGOS_615	FDAARGOS_759	NBRC 113806	82G9	CS1	CS2	CS4	CS5	CS6	CS7	CS8	CS12	CS13	CS15	CS17	CS18	CS20
**Capsular polysaccharide loci**	1	●	●			●		●	●	●	●	●	●	●	●	●	●	●	●	●	●		●	●	●		●
	2		●				●																			●	
	3	●																									
	4																										
	5	●	●	●	●				●	●	●	●	●	●		●								●			
	6																										
	7																										
	8	●	●		●	●	●		●	●	●	●		●				●		●							
	9				●	●									●					●							
	10																										
	11									●																	
	12																										
	13	●	●	●	●	●	●	●	●	●	●	●	●	●	●	●	●	●	●	●	●	●	●	●	●	●	●
	14																										

**Table 4:**
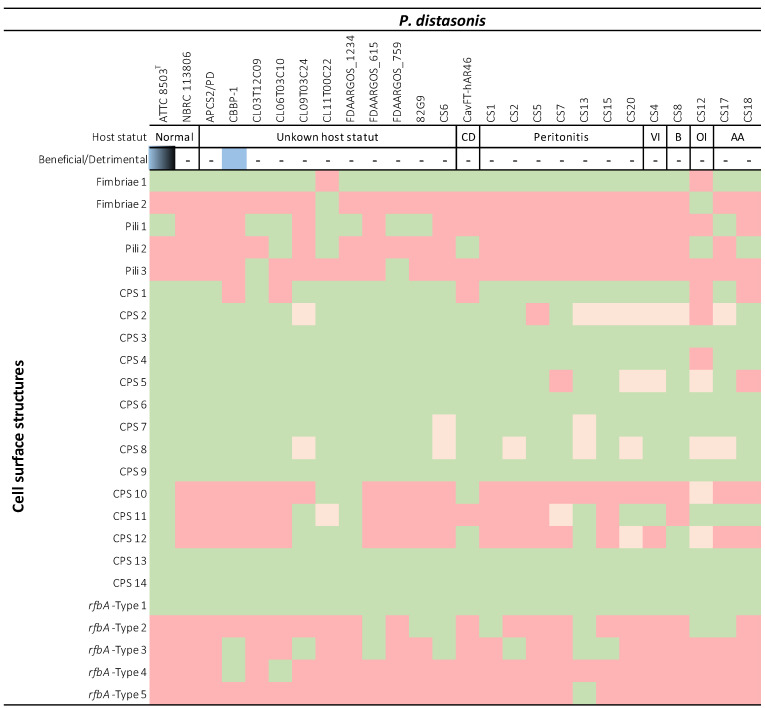

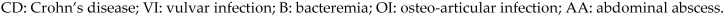
Identification of cell surface structures present on 26 *P. distasonis* strains based on host status. The beneficial or detrimental activity of strains (based on the literature) was added in order to compare potential pathogen from probiotic strains. Color code: beneficial properties (blue), detrimental properties (black), presence (green), partial presence (orange), absence (red). ATCC 8503^T^ is represented as blue/black for its beneficial/detrimental activities due to various results found in the literature. Dashes (-) have been added for unknown status.

**Table 5:** Reference genes used to determine external structures of *P. distasonis.*

Structure	Reference Genome	Gene	Label	Length (Aa)	Reference
**Capsule**	*Bacteroides fragilis* ATCC 25285^T^	*upaY*	BF1367	172	[11,42,55]
		*upaZ*	BF1368	157	
		*upbY*	BF1893	174	
		*upbZ*	BF1894	161	
		*upcY*	BF1009	172	
		*upcZ*	BF1010	130	
		*updY*	BF3699	179	
		*updZ*	BF3698	161	
		*upeY*	BF2606	172	
		*upeZ*	BF2605	160	
		*upfY*	BF1549	199	
		*upfZ*	BF1550	160	
		*upgY*	BF0731	178	
		*upgZ*	BF0732	162	
		*uphY*	BF3466	179	
		*uphZ*	BF3465	161	
**Fimbriae**	*Porphyromonas gingivalis* ATCC 33277^T^	*fimA*	PGN_0180	383	[12,15]
		*fimB*	PGN_0181	118	
		*fimC*	PGN_0183	462	
		*fimD*	PGN_0184	670	
		*fimE*	PGN_0185	550	
**Pilus**	*Bacteroides thetaiotaomicron* VPI-5482^T^	*mfa1*	BT_3147	388	[13]
		*mfa2*	BT_3148	430	
	*Porphyromonas gingivalis* ATCC 33277^T^	*mfa1*	PGN_0287	563	[14,33,48]
		*mfa2*	PGN_0288	324	
		*mfa3*	PGN_0289	446	
		*mfa4*	PGN_0290	333	
		*mfa5*	PGN_0291	1228	

## Data Availability

The genome sequencing data generated are indexed under the BioProject accession number PRJNA838851.

## Supplementary Materials

The following supporting information can be downloaded at: https://www.mdpi.com/article/10.3390/ijms23169411/s1.
