# Supplementary material for: In Silico Study of Cell Surface Structures of Parabacteroides distasonis Involved in Its Maintenance within the Gut Microbiota
Source: Int J Mol Sci. 2022 Aug 20;23(16):9411. doi: 10.3390/ijms23169411 (PMC9409006; doi:10.3390/ijms23169411)
Supplement: Supplementary file 1 [file ijms-23-09411-s001.zip › Supplementary data_Figure S1.pdf]

In Silico Study of Cell Surface Structures of *Parabacteroides distasonis* Involved in Its Maintenance within the Gut Microbiota and Its Potential Pathogenicity

Jordan Chamarande[1], Lisiane Cunat[1], Corentine Alauzet[1,2] and Catherine Cailliez-Grimal[1,*]

[1] SIMPA, Université de Lorraine, F-54000 Nancy, France
[2] CHRU de Nancy, Service de Microbiologie, F-54000 Nancy, France
[*] Correspondence: catherine.cailliez@univ-lorraine.fr

A

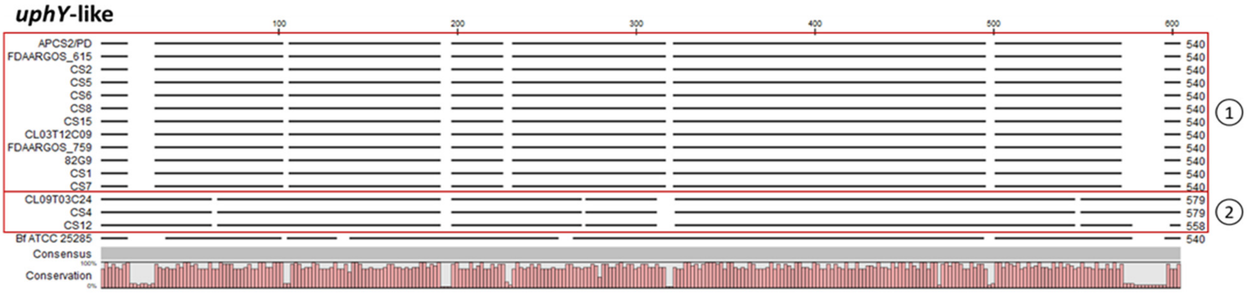
uphY-like
APCS2/PD
FDAARGOS_615
CS2
CS5
CS6
CS8
CS15
CL03T12C09
FDAARGOS_759
82G9
CS1
CS7
CL09T03C24
CS4
CS12
Bf ATCC 25285
Consensus
Conservation
540
579
558

B

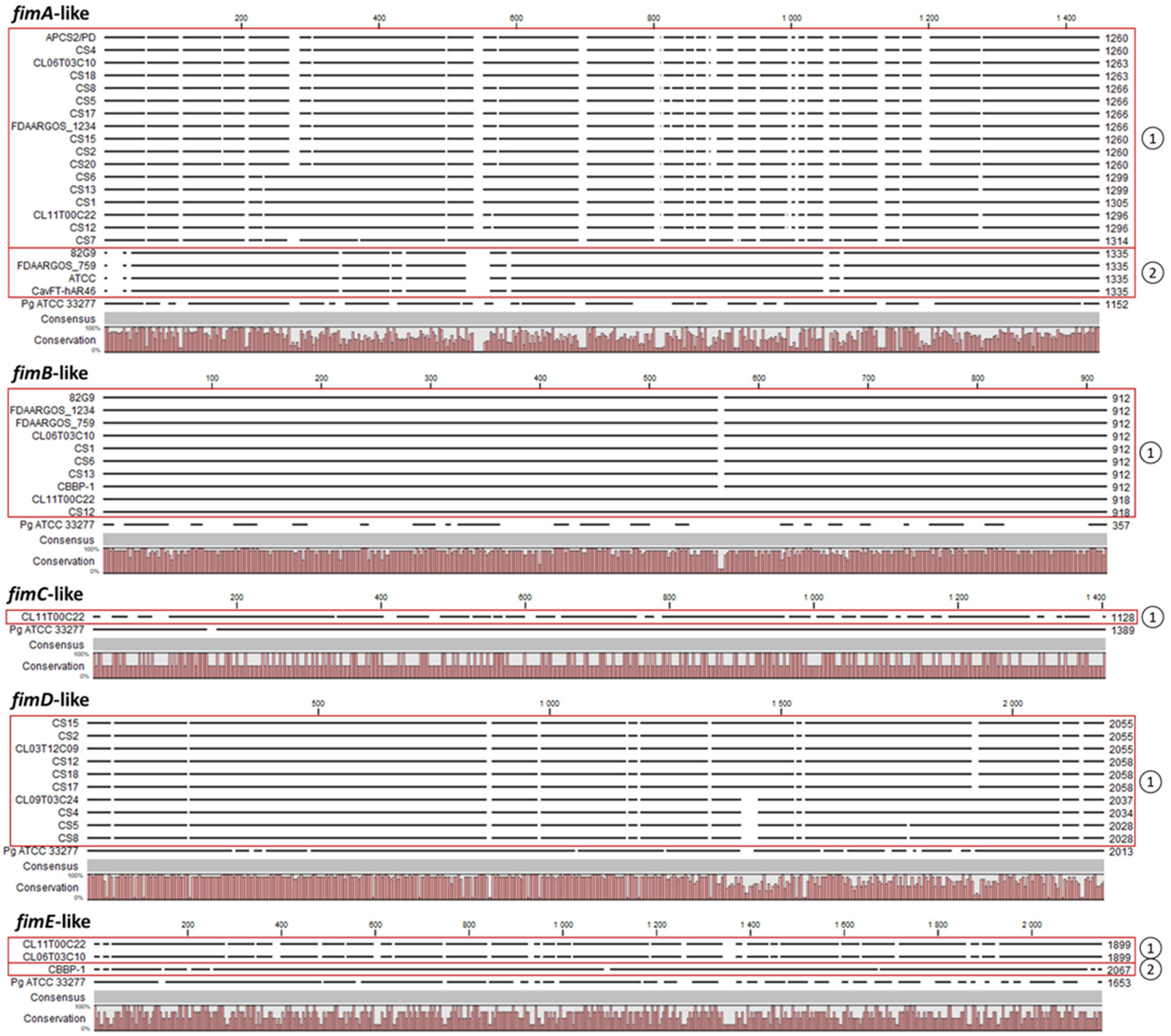
fimA-like
APCS2/PD
CS4
CL06T03C10
CS18
CS8
CS5
CS17
FDAARGOS_1234
CS15
CS2
CS20
CS6
CS13
CS1
CL11T00C22
CS12
CS7
82G9
FDAARGOS_759
ATCC
CavFT-hAR46
Pg ATCC 33277
Consensus
Conservation
fimB-like
82G9
FDAARGOS_1234
FDAARGOS_759
CL06T03C10
CS1
CS6
CS13
CBBP-1
CL11T00C22
CS12
Pg ATCC 33277
Consensus
Conservation
fimC-like
CL11T00C22
Pg ATCC 33277
Consensus
Conservation
fimD-like
CS15
CS2
CL03T12C09
CS12
CS18
CS17
CL09T03C24
CS4
CS5
CS8
Pg ATCC 33277
Consensus
Conservation
fimE-like
CL11T00C22
CL06T03C10
CBBP-1
Pg ATCC 33277
Consensus
Conservation

C

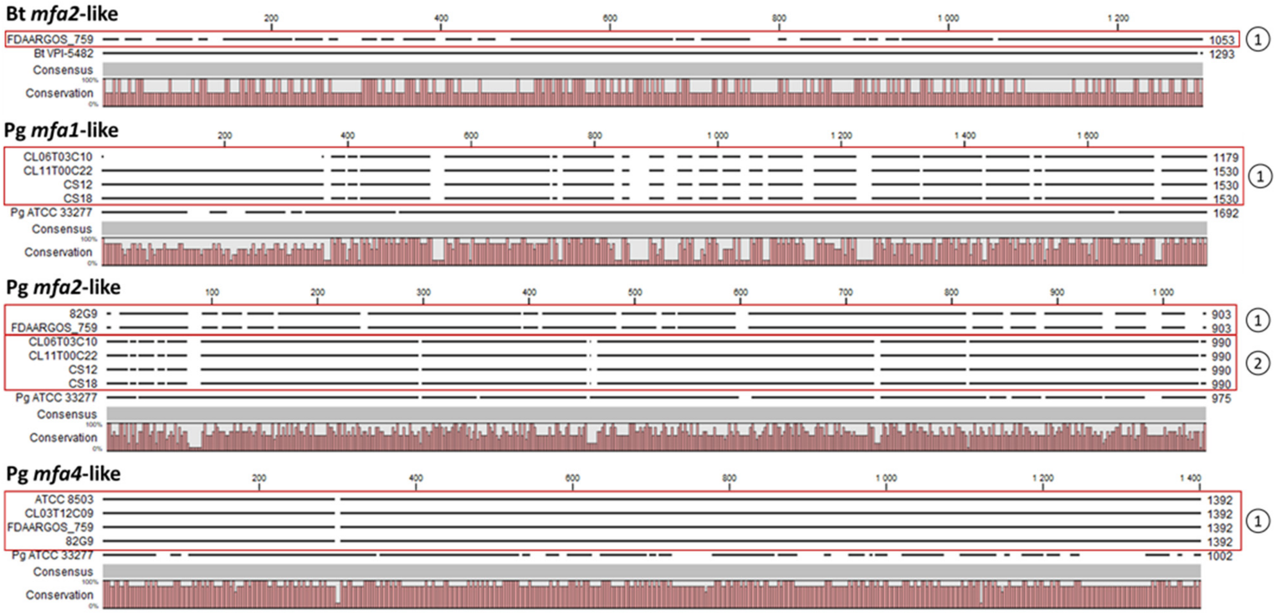

Figure S1. Multiple-sequence alignment of *P. distasonis* (**A**) *uphY*-like, (**B**) *fim*-like and (**C**) *mfa*-like genes listed in table 2. Each group of genes sharing similar patterns and sequences has been identified (red frame, 1 and 2) and aligned to generate a consensus sequence. As example, for *uphY*-like genes, two sequences have been generated: *uphY*-like 1 and *uphY*-like 2.
